# Jowiseungki decoction affects diabetic nephropathy in mice through renal injury inhibition as evidenced by network pharmacology and gut microbiota analyses

**DOI:** 10.1186/s13020-020-00306-0

**Published:** 2020-03-12

**Authors:** Xianglong Meng, Junnan Ma, Seok Yong Kang, Hyo Won Jung, Yong-Ki Park

**Affiliations:** 1 Department of Herbology, College of Korean Medicine, Dongguk University, Gyeongju, 38066 Korea; 2 Experimental Teaching Center, College of Chinese Materia Medica, Shanxi University of Chinese Medicine, Jinzhong, 030619 China

**Keywords:** Diabetic nephropathy, Jowiseungki decoction (JSD), Network pharmacology, Gut microbiota, PKCα/TGF-β1/Akt pathway, NF-κB/α-SMA pathway

## Abstract

**Background:**

Jowiseungki decoction (JSD) is a prescription commonly used for the treatment of diabetic complications or diabetic nephropathy (DN) in traditional medicine clinics. However, the underlying therapeutic mechanisms of JSD are still unclear.

**Methods:**

Streptozotocin (STZ)-induced DN mice were administered 100 and 500 mg/kg JSD for 4 weeks, and the therapeutic mechanisms and targets of JSD were analyzed by network pharmacology and gut microbiota analyses.

**Results:**

JSD significantly decreased the increase in food and water intake, urine volume, fasting blood glucose, serum glucose and triglyceride levels, and urinary albumin excretion. JSD administration significantly increased the decrease in insulin secretion and creatinine clearance and reduced the structural damage to the kidney tissues. Moreover, JSD administration significantly inhibited the expression of protein kinase C-alpha (PKC-α), transforming growth factor beta-1 (TGF-β1), α-smooth muscle actin (α-SMA), nuclear factor-κB (NF-κB), inducible nitric oxide synthase (iNOS), and cyclooxygenase-2 (COX-2) in the kidney tissues of DN mice, while it significantly increased the phosphorylation of insulin receptor substrate 1 (IRS-1), phosphatidylinositol-3-kinase (PI3K), and protein kinase B (Akt). In the network pharmacological analysis, JSD obviously influenced phosphatase binding, protein serine/threonine kinase, and mitogen-activated protein kinase (MAPK)-related signaling pathways. Our data suggest that JSD can improve symptoms in STZ-induced DN mice through the inhibition of kidney dysfunction, in particular, by regulating the PKCα/PI3K/Akt and NF-κB/α-SMA signaling pathways. Gut microbiota analysis can help to discover the pharmaco-mechanisms of the influence of JSD on bacterial diversity and flora structures in DN.

**Conclusion:**

JSD can improve the symptoms of DN, and the underlying mechanism of this effect is renal protection through the inhibition of fibrosis and inflammation. JSD can also change bacterial diversity and community structures in DN.

## Background

Diabetic nephropathy (DN) is the end-stage renal disease of type 2 diabetes mellitus (T2DM) [1], and the pathogenesis of DN is more complicated than that of other T2DM complications [2]. The treatment of T2DM and DN mainly includes the control of glucose levels using various hyperglycemic medicines and the management of hypertension and dietary salt intake, but many patients still progress to DN. Therefore, novel drugs for the treatment of DN or preventative strategies are needed [3].

According to the disease theories of Chinese and Korean traditional medicine, T2DM belongs to the wasting-thirst syndrome [4] characterized by yin vacuity with fire flaming upward, as well as qi vacuity. DN is the end stage of this syndrome with very serious renal dysfunction and excessive urination [5]. Therefore, therapeutic strategies, such as nourishing qi and yin, activating blood circulation and removing blood stasis, invigorating the spleen and kidney, clearing heat and relieving turbidity, are applied for DN treatment in traditional clinics. Jowiseungki decoction (JSD) was first used by Chang Chung-Ching in the Treatise on Febrile Disease (Shang Han Lun) and has been prescribed in traditional clinics in China (Tiaowei Chengqi decoction) and Korea (Jowiseungki decoction, JSD) for the treatment of T2DM, and its positive effects, including relieving constipation and clearing the internal heat in the stomach and intestine have been clinically suggested [6]. JSD is composed of three herbs, Rhubarb (*Rheum palmatum*, L*., Rheum officinale* Baill., *Rheum tanguticum* Maxim, ex Balf.), Mirabilitum, and licorice (*Glycyrrhizae uralensis* Fisch., *Glycyrrhiza inflata* Bat*., Glycyrrhiza glabra* L.). Rhubarb and Mirabilitum are cold-natured herbs in herbology and are applied to control inflammation [7, 8]. Thus, their anti-inflammatory effects have been experimentally proven in both in vitro and in vivo studies [7–9]. Liquorice is a calming and sweet-natured herb, and its protective antioxidant effects on liver injuries have been reported [7]. Although JSD is a well-known prescription for DM in traditional medicines, the mechanisms responsible for its effects in experimental studies, including preclinical studies, are poorly understood.

Meanwhile, to modernize traditional medicine, new analytical methods, such as network pharmacology and gut microbiota analysis, have recently been introduced. Network pharmacology offers a new research paradigm from the current “one target and one drug” mode to a new “network target and multicomponent” mode [8]. Moreover, network-based pharmacological analysis can provide insight into the active mechanisms of individual herbs or herbal prescriptions by providing information about their potential bioactive components at the molecular and systematic levels [10]. According to traditional medicine theories, the human body and the external environment are an organic whole, and the unity of the internal and external environment is considered the overall goal. The unified theory of biology and environment is the common theoretical basis shared by holistic medicine and microecology. Currently, the interaction of intestinal flora and pharmacodynamic substances has attracted increasing attention in traditional medicine research. Recent studies have found that intestinal flora can significantly regulate the secretion of insulin [11], glucagon and other hormones [12] and play an important role in the development of insulin resistance [13], which can reveal scientific applications of traditional medicine symptoms.

Therefore, in this study, we investigated the therapeutic effects of JSD on streptozotocin (STZ)-induced DN mice and the responsible mechanism, with a particular focus on renal dysfunction. We also analyzed the main compounds in JSD and discovered their molecular targets and functions using network pharmacology and gut microbiota analysis.

## Methods

### The preparation of Jowiseungki extract

All JSD herbs (Table 1) were purchased from Kwangmyungdang Medicinal Herbs (Ulsan, Korea) and verified by Professor Yong-Ki Park, a medical botanist in the College of Korean Medicine, Dongguk University. The herbs were mixed to a total of 196 g, extracted in 1.96 l of boiling water for 3 h, filtered through Whatman paper filter No. 1 (Maidstone, UK), concentrated using a rotating decompressor (Eyela, Tokyo, Japan) and freeze dried (ilShinBioBase, Yangju, Korea). The final yield of JSD was 53.92%.

**Table 1:** The composition of JSD

Common name	Scientific name (Family)	Herb name	Ratio	Weight (g)
Rhubarb	*Rheum palmatum,* L. *Rheum officinale* Baill. *Rheum tanguticum* Maxim, ex Balf.	Rhei Radix et Rhizoma	4	112
Mirabilitum	–	Natrii Sulfas	2	56
Liquorice	*Glycyrrhizae uralensis* Fisch. *Glycyrrhiza inflata* Bat. *Glycyrrhiza glabra* L.	Glycyrrhizae Radix et Rhizoma	1	28

### HPLC analysis of JSD

To analyze the constituents of JSD, we performed high-performance liquid chromatography (HPLC) using two chromatographic conditions (14,15). The mobile phase for liquiritin and ammonium glycyrrhizate consisted of deionized water with 0.1% phosphoric acid (A) and acetonitrile (B), and the gradient elution program was as follows: 19% B, 0–8 min; 19–50% B, 8–35 min; 50–100% B, 35–36 min; and 100–19% B, 36–40 min. The detection wavelength was set at 237 nm. The mobile phase for aloe-emodin, rheinic acid, rheum emodin, chrysophanol, and emodin-3-methyl ether consisted of deionized water with 0.1% phosphoric acid (A) and methanol (B), and the isocratic elution program was as follows: 85% B, 0–25 min. The detection wavelength was set at 254 nm. All HPLC analyses were performed with a U3000 series system (Thermo Fisher Scientific, Waltham, MA, USA) and were then separated on Tnature C18 (250 × 4.6 mm, 5 μm, Waters Co., Ltd., Trenton, NJ, USA). The HPLC pattern of the components in JSD has been reported in the literature [14]. The flow rate was 1.0 mL/min, the injection volume was 10 μL, and the column temperature was maintained at 30 °C. The liquiritin, ammonium glycyrrhizate, aloe-emodin, rheinic acid, rheum emodin, chrysophanol, and emodin-3-methyl ether standards (production batch numbers 18060604, 18083102, 18032708, 18010901, 18022605, 18011903, and 17110101, respectively) were purchased from Chengdu Pufei De Biotech Co., Ltd. (Chengdu, China). The mass fraction of all the standard reagents was ≥ 98%.

### Animals and preparation of diabetic animal model

Five-week-old male specific pathogen-free (SPF) C57B/6 mice (18–20 g) were purchased from Orient Bio Inc. (Seongnam, Korea). The mice were acclimated to the SPF laboratory for 1 week before the experiment began. The mice were raised under a 12 h light/12 h dark cycle at 23 ± 2 °C and 50 ± 10% humidity and were given filtered water. All animals were handled according to the animal welfare guidelines issued by the Korean National Institute of Health and the Korean Academy of Medical Sciences for the care and use of laboratory animals and approved by the Institutional Animal Care and Use Committee of Dongguk University (IACUC-2017-012).

**Fig. 1:**
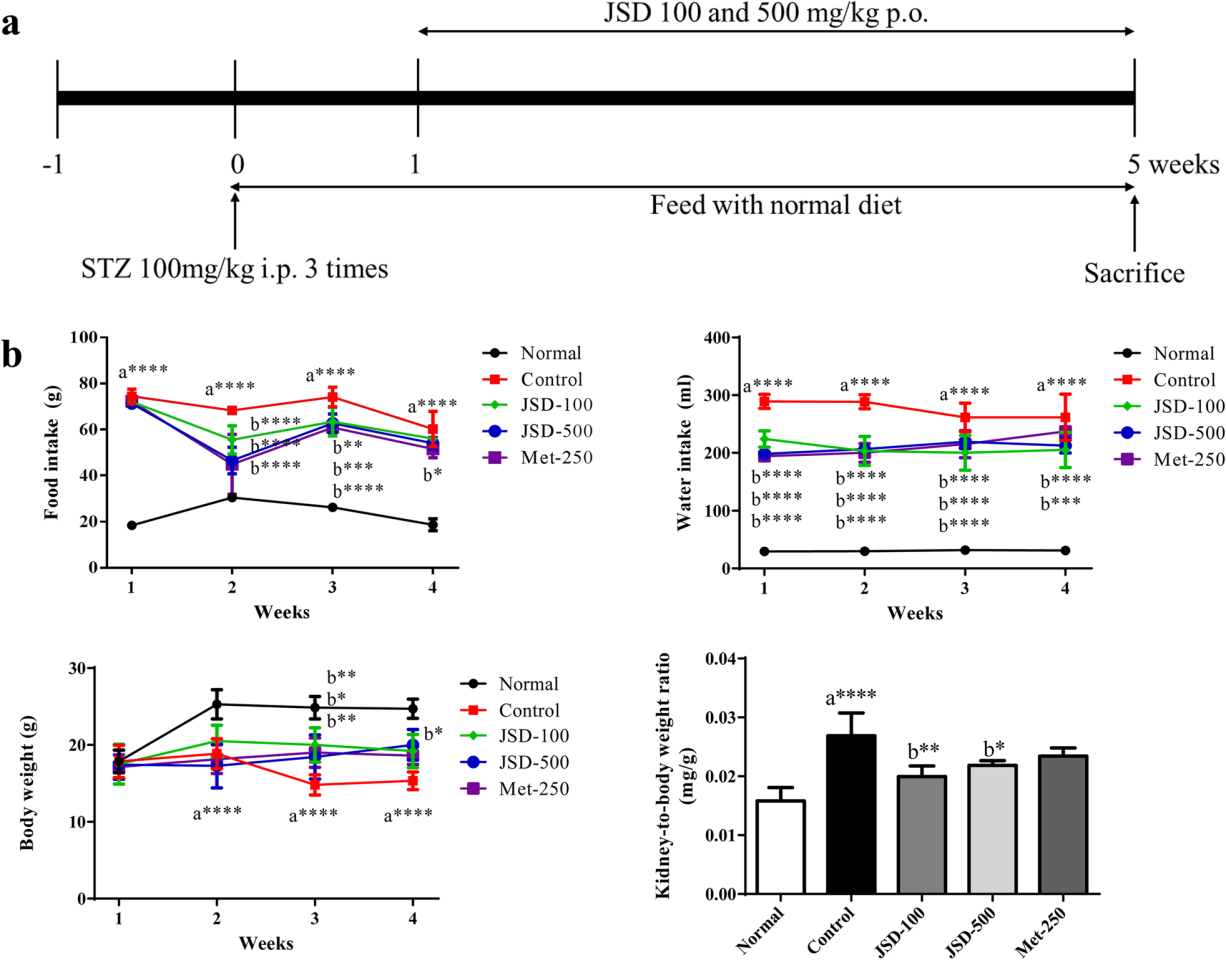
Effects of JSD on the physiological changes in DN mice. **a** Experimental design of the DN mouse model. The mice were allowed to adapt for 1 week (− 1 W to 0 W). STZ at 100 mg/kg of body weight was injected (i.p.) once per day for 3 consecutive days to induce DN (0 W to 1 W). JSD (100 and 500 mg/kg) and metformin (250 mg/kg) were orally administered to the assigned groups for 4 weeks (1 W to 5 W). Body weight, food and water intake and fasting blood glucose level were measured once per week. All mice were sacrificed at the end of 5 W, and blood and tissues were collected for further analysis. **b** Effects of JSD on the physiological changes in DN mice. Normal, normal group; Control, STZ-induced DN control group; JSD-100, 100 mg/kg JSD-administered group; JSD-500, 500 mg/kg JSD-administered group; and Met-250, 250 mg/kg metformin-administered group. Data are expressed as the mean ± S.D. (n = 7 per group); *p < 0.05, **p < 0.01 and ***p < 0.001, the control group vs. the normal group (**a**) and the JSD-100, JSD-500 and Met-250 groups vs. the control group (**b**)

To induce DN in mice, STZ at 100 mg/kg of body weight (b.w.) (Sigma-Aldrich, St. Louis, MO, USA) was injected (i.p.) once per day for 3 consecutive days. All mice were randomly divided into the following five groups (n = 7 per group): normal group, STZ-induced diabetic control group, JSD (100 and 500 mg/kg b.w.)-administered groups ((p.o.), and metformin (250 mg/kg)-administered group (Met, p.o.), as a reference group. All mice were given a standard diet (3.1 kcal, 14% protein, Harlan Teklad, Madison, WI, USA) for the whole experimental period (Fig. 1a). After 4 weeks of administration, all mice were fasted for 12 h and then sacrificed. Blood and kidney samples were harvested for further analysis. The intestinal feces were taken under sterile conditions, and then put into sterile tubes in liquid nitrogen.

### Measurement of physiological characteristics

The changes in the physiological parameters-body weight, water and food intake, and fasting blood glucose (FBG) level-were measured once per week for 4 weeks. In addition, 24 h urine volumes were collected from all mice in metabolic cages at the end of the experimental period.

### Measurement of serological and urine markers

A commercial ELISA kit (catalog # 90080; Crystal Chem, Springfield, IL, USA) was used to measure the insulin concentration in the serum, according to the manufacturer’s protocol. The levels of aspartate aminotransferase (AST), alanine aminotransferase (ALT), glucose, total cholesterol (TC), triglyceride (TG), HDL-cholesterol (HDL-C), urea nitrogen (UN), creatinine (Cr), and microalbumin/urine creatine (MA/UCREA) were measured in the serum or urine samples using an automatic biochemistry analyzer (Fuji Dri-chem 700i, Fujifilm, Tokyo, Japan).

### Western blot

Kidney tissues were isolated from all mice, and the protein was extracted using the T-PER tissue protein extraction reagent (Thermo Fisher Scientific, Waltham, MA, USA). The protein content was measured using Bradford’s assay, and 30 μg of protein was separated via SDS-PAGE and transferred to a nitrocellulose membrane. The membrane was incubated in 5% skim milk in Tris-buffered saline with 1% Tween-20 (TBST) (pH 7.5) for 30 min at room temperature (RT) for blocking and then incubated with antibodies against PKC-α, I-κB, NF-κB(p 65), α-SMA, TGF-β1, COX-2 (Santa Cruz Biotechnology, Santa Cruz, Dallas, TX, USA), iNOS (BD Biosciences, San Jose, CA, USA), total-AKT(Ser473), phospho-AKT(Ser473), total-IRS(Ser 307), phospho-IRS(Ser 307), PI3K (p85) (Cell Signaling Technology, Danvers, MA, USA), and β-actin (Sigma-Aldrich, Inc., St. Louis, MO, USA) overnight at 4 °C. The membrane was washed three times with TBST and incubated with HRP-conjugated mouse or rabbit secondary antibodies for 3 h at room temperature. The expression of each protein target was detected by the ChemiDoc™ MP Imaging system (Bio-Rad Laboratories, USA), and the relative ratio of each target to β-actin was analyzed using an analysis program.

### Quantitative PCR assay

Total RNA was isolated from kidney tissues using NucleoZOL reagent (Macherey–Nagel GmbH & Co. KG., Duren, Germany) according to the manufacturer’s instructions. RNA concentration was measured using a spectrophotometer (NanoDrop Technologies, Inc., Wilmington, Delaware, USA). cDNA was generated with 1 μg of total RNA using a Reverse Transcription System kit (Promega, Fitchburg, Wisconsin, USA). PCR was performed using a SYBR Green kit (Agilent Technologies, West Cedar Creek, TX, USA) and primers specific to the target genes (Table 2). Gene expression was evaluated by the ΔΔCT method, using GAPDH as a housekeeping gene.

**Table 2:** Primer sequences for RT-PCR

Primers	Accession no.	Sequence (5′–3′)
PKC-α		
Forward	NM_011101.3	CCCATTCCAGAAGGAGATGA
Reverse	NM_011101.3	TTCCTGTCAGCAAGCATCAC
α-SMA		
Forward	XM_006526606.2	ACTGCCGAGCGTGAGATTGT
Reverse	XM_006526606.2	TGATGCTGTTATAGGTGGTTTCG
TGF-β1		
Forward	NM_011577.2	CGAAGCGGACTACTATGCTAAAGAG
Reverse	NM_011577.2	TGGTTTTCTCATAGATGGCGTTG
GAPDH		
Forward	XM_017321385.1	CAGCCTCGTCCCGTAGACA
Reverse	XM_017321385.1	CGCTCCTGGAAGATGGTGAT

### Histopathological observation

The kidney tissues were isolated, fixed in 4% paraformaldehyde solution for 48 h, processed in 10–30% sucrose solution, dehydrated in a graded alcohol series, and then embedded in paraffin wax. The paraffin tissues were cut into 4 μm thick sections by a cryotome and were stained with hematoxylin and eosin (H&E), periodic acid–Schiff (PAS), or Masson’s trichrome (M-T). The morphological changes in the stained tissues were observed under a light microscope.

### Network construction and analysis of the effect of JSD on DN

The chemical ingredients of JSD were obtained from the Traditional Chinese Medicine Systems Pharmacology (TCMSP) database (http://lsp.nwu.edu.cn/tcmsp.php), the Bioinformatics Analysis Tool for the Molecular Mechanism of Traditional Chinese Medicine (http://bionet.ncpsb.org/batman-tcm/index.php), and associated literature. In view of complexity of the pharmacodynamic chemical components in the Chinese herbal formula, the compounds were screened for both pharmacokinetic and pharmacodynamic properties (oral bioavailability (OB) > 30% and drug-likeness (DL) > 0.18%). The related DN targets were identified from Genecards (http://www.genecards.org) and the Online Mendelian Inheritance in Man (OMIM) database (http://www.omim.org). An integrative analysis of JSD, the directly related genes, and the DN targets was performed using a Venn analysis. The overlap was presumed to be the group of potential targets that were used in the network pharmacological analysis. An interaction network was established for the active ingredients, putative targets, and DN-associated targets of JSD. The interaction network was visualized by Cytoscape 3.7.1 software (https://cytoscape.org).

### Pathway and functional enrichment analysis of the effect of JSD on DN

We obtained the protein–protein interaction network (PPI network) by using STRING 11.0 (https://string-db.org/cgi/input.pl). We also performed a Gene Ontology (GO) functional enrichment analysis using the Database for Annotation, Visualization, and Integrated Discovery (https://david.ncifcrf.gov), and the Kyoto Encyclopedia of Genes and Genomes (KEGG) (http://www.genome.jp/kegg) Pathway Database was chosen for the pathway enrichment analysis of JSD.

### Gut microbiota analysis

Feces were collected from all mice on the fifth week for gut microbiota analysis. Mouse fecal samples were randomly selected from each group and thawed at 4 °C on ice. Then, 30 ng was used for PCR amplification after centrifugation, fully mixing and quality inspection by Nanodrop. The parameters of agarose gel electrophoresis were as follows: gel concentration, 1%; voltage, 170 V; electrophoresis times, 30 min. Basic quality control analysis, alpha diversity analysis, operational taxonomic unit (OTU) clustering analysis, and alpha and beta diversity analysis were conducted successively.

### Statistical analysis

The results of the experiment were statistically analyzed, including the calculation of the mean and standard deviation (mean ± SD), using ImageJ (version 1.4, LOCI, Madison, WI, USA) and GraphPad Prism 5.0 (GraphPad Software, La Jolla, CA, USA). Differences between groups were considered significant when the confidence interval was equal to 95% using one-way ANOVA and Tukey’s test.

## Results

### Effects of JSD on the physiological changes in DN mice

To investigate the effects of JSD on the physiological changes in STZ-induced DN mice, we measured body weight (BW) and food and water intake once per week for four weeks and kidney weight on the last day of the experiment. As shown in Fig. 1b, a significant difference (p < 0.0001) was found between the normal and DN control groups in food and water intake, BW, and the ratio of kidney weight to BW.

JSD administration at low (100 mg/kg) and high (500 mg/kg) doses in DN mice significantly reduced the increase in food intake (*p* < 0.0001 in the second week and *p* < 0.01 in the third week for the low dose, and *p* < 0.0001 in the second and the third week for the high dose) and water intake (*p* < 0.0001 throughout the period for the low dose and *p* < 0.0001 from the first to the third week and *p* < 0.001 in the fourth week for the high dose) compared with the nontreated group. Additionally, the metformin-administered group showed significantly decreased BW (*p* < 0.05 in the third week), food intake (*p* < 0.0001 int the second and third weeks and *p* < 0.05 in the fourth week), and water intake (*p* < 0.0001 from the first to the third week) in DN mice.

Moreover, compared with the nontreated control condition, the administration of JSD to DN mice significantly increased the decrease in BW in the third week (*p* < 0.001 for the low dose and *p* < 0.05 for the high dose).

With respect to the changes in the ratio of kidney weight to BW, a significant increase in kidney weight (p < 0.0001) was observed in the DN control group compared with the normal group, but the decrease in kidney weight was significantly decreased in the JSD-administered group (*p* < 0.01 for the low dose and *p* < 0.05 for the high dose) compared with the control group.

### Effects of JSD on serological changes in DN mice

To investigate the effects of JSD on DN, we measured serological markers, such as fasting blood glucose (FBG) and serum glucose, insulin, lipid, AST, and ALT levels, in mice after 4 weeks, on the last day of the experiment.

**Fig. 2:**
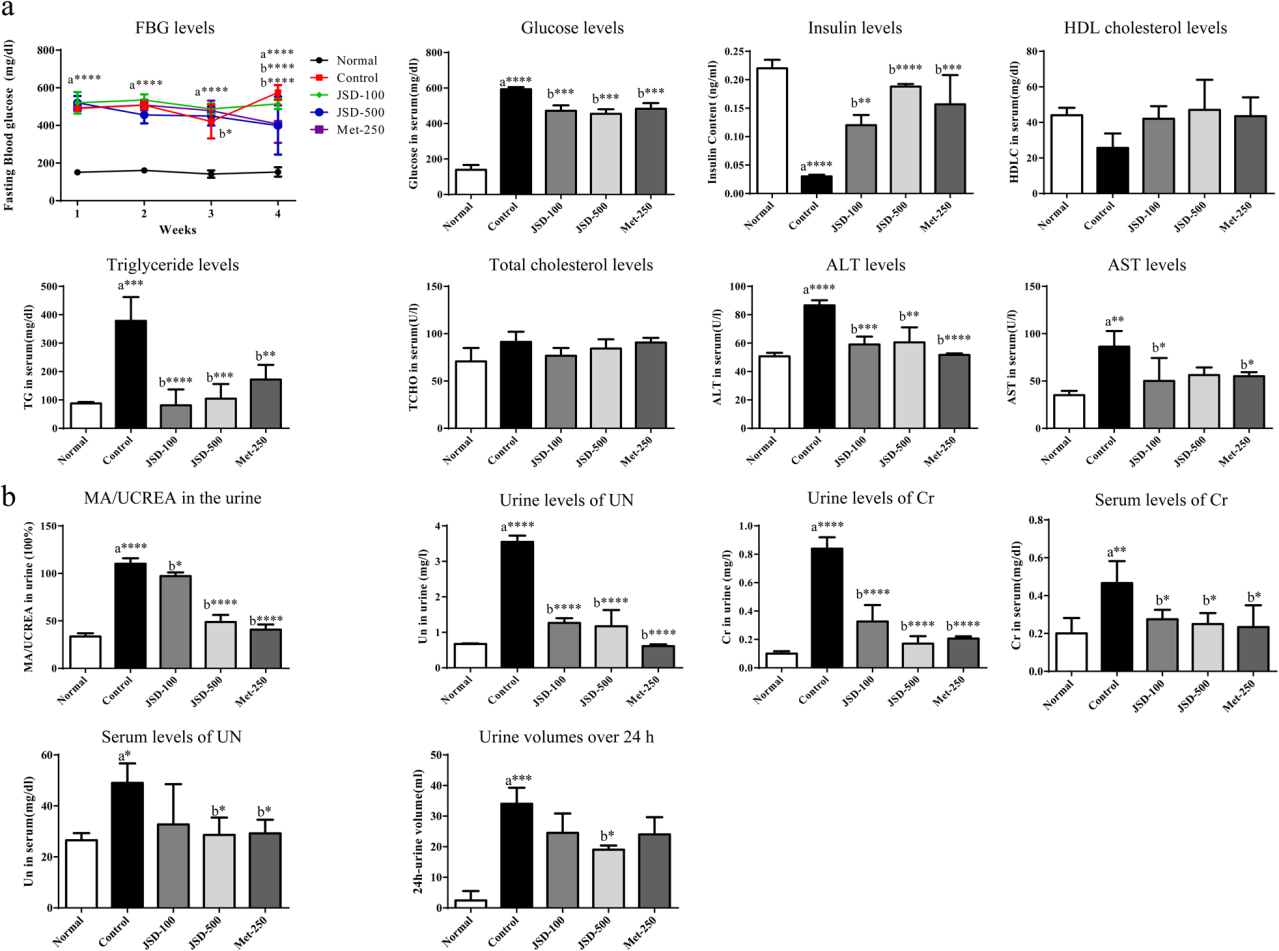
Effects of JSD on the serological changes (**a**) and kidney dysfunction (**b**) in DN mice. Normal, normal group; Control, STZ-induced DN control group; JSD-100, 100 mg/kg JSD-administered group; JSD-500, 500 mg/kg JSD-administered group; and Met-250, 250 mg/kg metformin-administered group. Data are expressed as the mean ± S.D. (*n *= 7 per group); *p < 0.05, **p < 0.01 and ***p < 0.001, the control group vs. the normal group (**a**) and the JSD-100, JSD-500 and Met-250 groups vs. the control group (**b**)

As shown in Fig. 2a, a significant increase in FBG was observed in the DN control group (*p* < 0.0001 in each week) compared with the normal group, and this increase was significantly decreased by the administration of JSD (*p* < 0.05 for the low dose in the third week and *p* < 0.0001 for the high dose in the fourth week). The metformin-treated groups also showed a significant decrease in FBG levels compared with the DN control group (*p* < 0.0001 in the fourth week).

Compared with the control condition, the administration of JSD significantly reduced the serum glucose levels at both low (*p* < 0.001) and high (*p* < 0.001) doses. JSD administration significantly increased insulin levels at low (*p* < 0.01) and high (*p* < 0.0001) doses. Metformin also significantly decreased glucose levels (*p* < 0.001) and significantly increased insulin levels (*p* < 0.001).

Regarding the lipid profile, compared with the nontreated control condition, the administration of JSD at low and high doses to DN mice increased HDL-cholesterol levels and decreased total cholesterol and triglyceride levels (*p* < 0.0001 for the low dose and *p* < 0.001 for the high dose).

For liver damage, the serum levels of AST (*p* < 0.0001) and ALT (*p* < 0.01) were significantly increased in DN mice compared with control mice. The JSD-treated group showed significantly decreased levels of AST (*p* < 0.05 for the low dose) and ALT (*p* < 0.001 for the low dose and *p* < 0.05 for the high dose) compared with the DN control group. Metformin also significantly reduced the serum levels of AST (*p* < 0.05) and ALT (*p* < 0.0001) in DN mice.

### Effects of JSD on kidney dysfunction in DN mice

To investigate the effects of JSD on kidney dysfunction in DN, we measured the ratio of microalbumin and creatine (MA/UCREA) in urine, the levels of urea nitrogen (UN) and creatinine (Cr) in urine and serum, and urine volumes for 24 h on the last day of the experiment (Fig. 2b).

A significant increases in the MA/UCREA ratio (*p* < 0.0001), UN (*p* < 0.0001), and Cr (*p* < 0.0001) in urine were observed in the DN control group compared with the normal group. Compared with the control condition, the administration of JSD at low (*p* < 0.05) and high (*p* < 0.0001) doses for 4 weeks significantly decreased the ratio of MA/UCREA. In addition, JSD administration significantly decreased the levels of UN and creatinine at low (*p* < 0.0001, respectively) and high (*p* < 0.0001, respectively) doses in DN mice. Compared with the control condition, metformin treatment was also shown to significantly decrease the ratio of MA/UCREA and the levels of UN (*p* < 0.0001) and Cr (*p* < 0.0001).

Significant increases in serum UN (*p* < 0.01) and Cr (*p* < 0.05) were shown in the DN control group compared with the normal group. The administration of JSD to DN mice significantly reduced the levels of UN (*p* < 0.05 for the high dose) and Cr (*p* < 0.05 for the low and high dose). Compared with the control group, the metformin-treated group also showed significantly decreased UN (*p* < 0.05) and Cr (*p* < 0.05) levels in the serum.

A significant increase in 24-h urine volume (*p* < 0.001) was found in the DN control group compared with the untreated control group, while JSD administration at the high dose significantly (*p* < 0.05) decreased the increasing trend in urine volume in DN mice.

### Effects of JSD on histopathologic changes in kidney tissues in DN mice

**Fig. 3:**
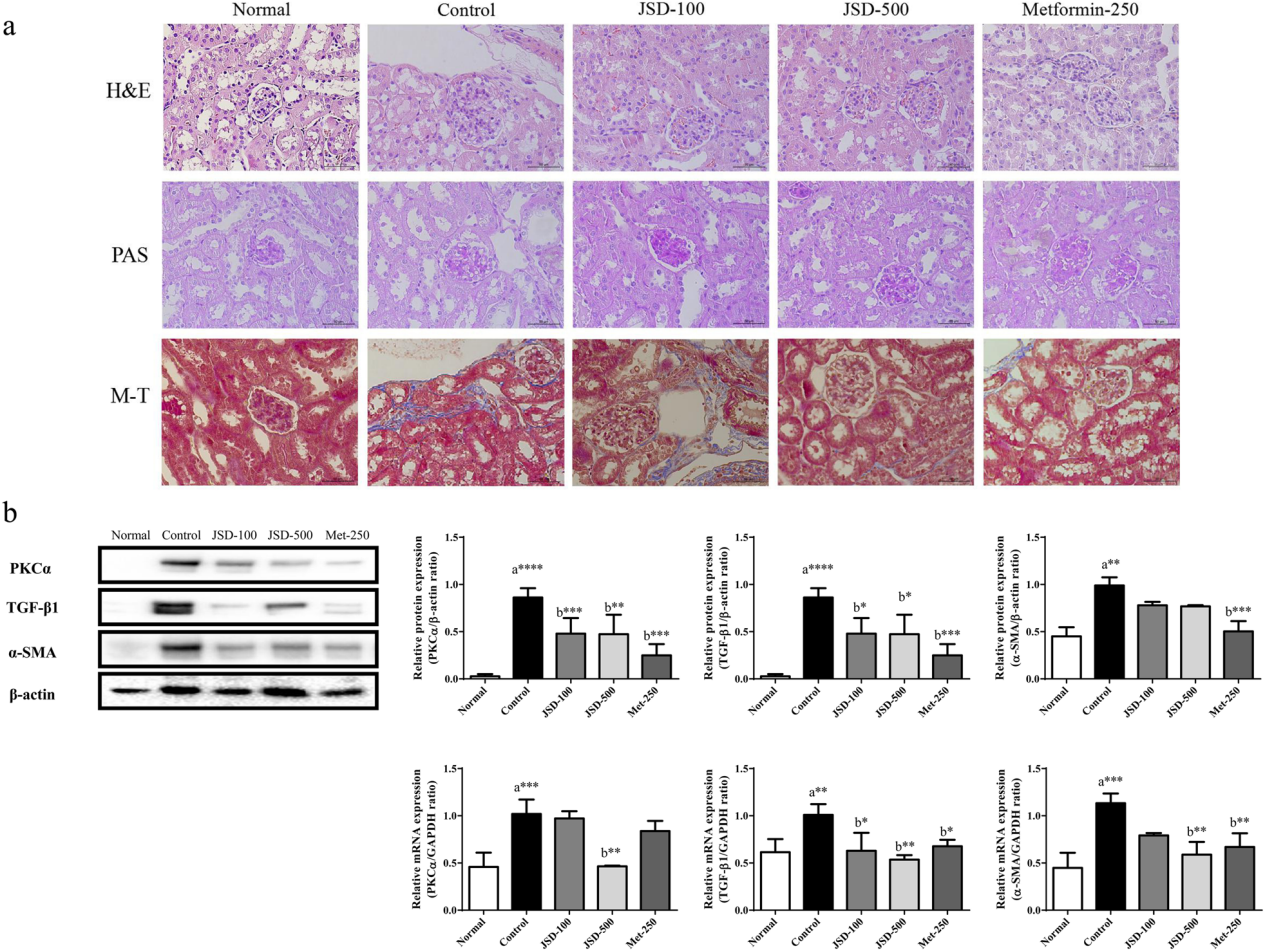
Effects of JSD on the histological changes (**a**) and the expression of fibrosis regulators (**b**) in kidney tissues in DN mice. All tissues were observed under a light microscope (× 400), and representative pictures were shown. Relative levels of PKCα, TGF-β1 and α-SMA proteins were normalized to β-actin expression and determined by western blotting. Relative levels of PKCα, TGF-β1 and α-SMA mRNA were normalized to GAPDH expression and determined by reverse transcription-quantitative polymerase chain reaction analysis. Normal, normal group; Control, STZ-induced DN control group; JSD-100, 100 mg/kg JSD-administered group; JSD-500, 500 mg/kg JSD-administered group; and Met-250, 250 mg/kg metformin-administered group. Data are expressed as the mean ± S.D. (n = 7). *p < 0.05, **p < 0.01 and ***p < 0.001, the control group vs. the normal group (**a**)and the JSD-100, JSD-500 and Met-250 groups vs. the control group (**b**)

Next, we observed histological changes in the kidney using H&E, PAS, and Masson’s trichrome (M-T) staining of kidney tissues (Fig. 3a). In the normal group, well-opened glomerular capillary loops without inflammatory cells were observed with H&E, PAS, and M-T staining. In the DN control group, H&E staining revealed increased volumes of glomerular loops with nodular sclerosis and mesangial stromal hyperplasia. The PAS stain revealed an obvious mesangial matrix expansion. The M-T stain showed an increased fibrosis intensity in response to the accumulation of the extracellular matrix proteins in the control group. However, these destructive changes mediated by DN induction were markedly ameliorated by JSD administration at low and high doses and by metformin. These results indicate that JSD has a protective effect against renal damage in DN mice.

### Effects of JSD on the expression of fibrosis regulators in the renal tissues of DN mice

To investigate the protective effects of JSD on kidney damage, in particular renal fibrosis (a main symptom of DN), we evaluated the mRNA and protein (Fig. 3b) expression of renal fibrosis regulating factors (PKC-α, TGF-β1, and α-SMA by both quantitative PCR and western blot analysis.

The expression levels of PKC-α (*p* < 0.0001 for protein and *p* < 0.001 for mRNA), TGF-β1 (*p* < 0.0001 for protein, *p* < 0.01 for mRNA), and α-SMA (*p* < 0.01 for protein and *p* < 0.001 for mRNA) in kidney tissues were significantly increased in the DN control group compared with the normal group. Moreover, JSD administration significantly inhibited the expression of the PKC-α, TGF-β1, and α-SMA protein (*p* < 0.001 for the low dose and *p* < 0.01 for the high dose for PKC-α, *p* < 0.05 for TGF-β1 for both doses) and mRNA (*p* < 0.01 for the high dose for PKC-α, *p* < 0.05 for the low dose and *p* < 0.01 for the high dose for TGF-β1, and *p* < 0.01 for the high dose for α-SMA) compared with the control group. Metformin administration also significantly decreased the expression of the PKC-α, TGF-β1, and α-SMA protein (*p* < 0.001) and mRNA (*p* < 0.05 for TGF-β1 and *p* < 0.01 for α-SMA).

### HPLC analysis of JSD

**Fig. 4:**
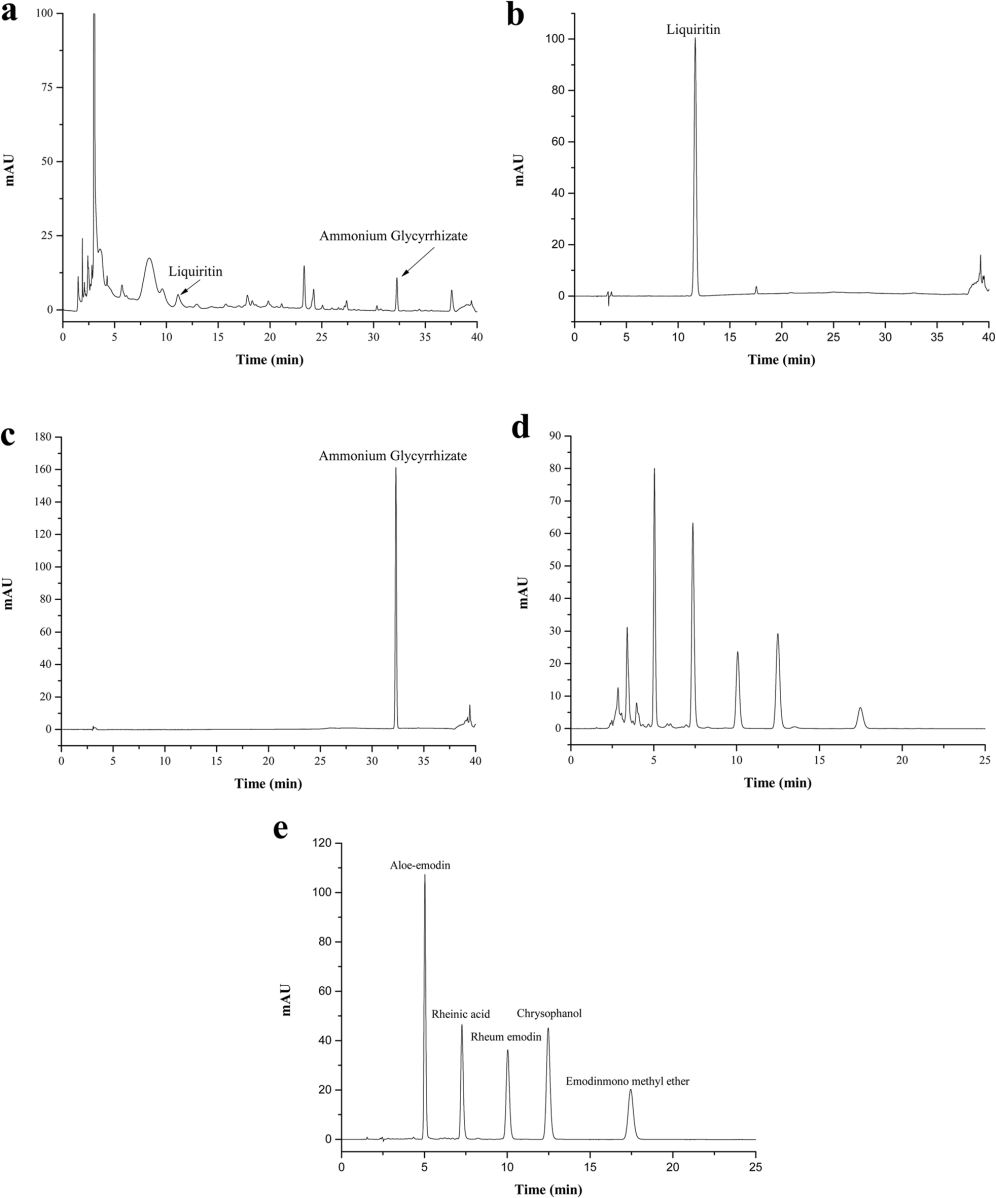
HPLC profiles of JSD. HPLC patterns of JSD extract at 237 nm (**a**), 254 nm (**d**) and standard compounds, liquiritin, ammonium glycyrrhetate at 237 nm (**b**, **c**), aloe-emodin, rheinic acid, rheum emodin, chrysophanol, and emodin-3-methyl ether at 254 nm (**e**)

To identify the main compounds in JSD, we performed an HPLC analysis using the standard compounds (Table 3) in each constituent (Table 1) reported in previous literature (10,11,26). In our analysis, seven compounds, liquiritin and ammonium glycyrrhizate in Liquorice (Fig. 4a–c) and aloe-emodin, rheinic acid, rheum emodin, chrysophanol, and emodin-3-methyl ether in Rhubarb (Fig. 4d, e), showed peaks in the liquid chromatogram. The calibration curve equations for the standard compounds were as follows: Y = 76.6945X + 1.8357 (r = 0.9995) for liquiritin, Y = 42.7008X + 0.3506 (r = 0.9991) for ammonium glycyrrhizate, Y = 505.2X + 0.4877 (r = 0.9997) for aloe-emodin, Y = 288.59X + 0.532 (r = 0.9998) for rheinic acid, Y = 463.13X − 0.0302 for rheum emodin, Y = 762.19X + 0.0778 (r = 0.9997) for chrysophanol, and Y = 456.83X + 0.0269 (r = 0.9998) for emodin-3-methyl ether.

**Table 3:** Contents of the main compounds in JSD

Component	Concentration (mg/g)
Liquiritin	1.6335 ± 5.902 × 10^−2^
Ammonium glycyrrhizate	1.5592 ± 1.617 × 10^−2^
Aloe-emodin	1.9940 ± 3.121 × 10^−2^
Rheinic acid	5.6548 ± 3.223 × 10^−2^
Rheum emodin	1.5873 ± 3.383 × 10^−2^
Chrysophanol	1.2897 ± 2.408 × 10^−2^
Emodin-3-methyl ether	1.2302 ± 1.161 × 10^−2^

### Analysis and verification of the functional network of JSD in DN

To explore the potential ingredients and molecular targets of JSD that affect DN, we conducted a network pharmacological analysis. In our analysis, we discovered 111 potential active compounds in JSD, including 18 compounds in Rhubarb, 92 compounds in Liquorice, and one compound in Mirabilitum (Natrii Sulfas). We also searched 2172 DN targets and collected 220 targets corresponding to those effective constituents of JSD in DN. We summarized the final 126 targets of JSD that were ascertained to have a close association with DN after establishing the Venn intersection targets (Fig. S1 in Additional file 1).

**Fig. 5:**
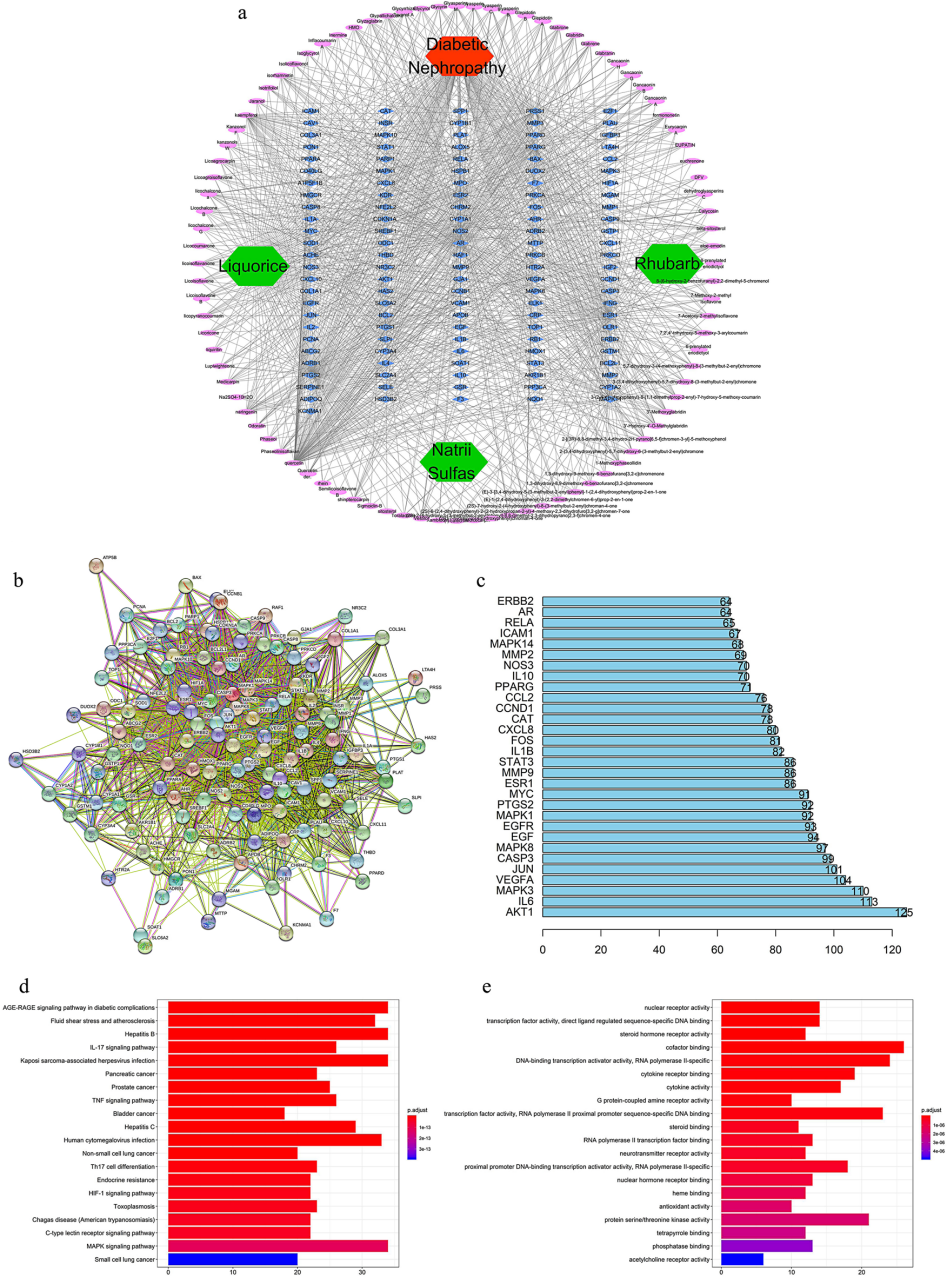
Network construction and pathway and functional enrichment analysis of the effect of JSD on DN. **a** Potential active ingredient-target-disease network. The different colors of the symbols represent the following: disease (red), herb names (green), targets (blue) and compounds (purple). **b** PPI network. The following nodes are shown: query proteins and direct interactors (colored nodes), secondary interactors (white nodes), proteins with unknown 3D structure (empty nodes), proteins with known or predicted 3D structure (filled nodes) from curated databases ( ), experiments ( ), gene neighborhood ( ), gene fusions ( ), gene cooccurrence ( ), text mining ( ), coexpression ( ), and protein homology ( ). **c** Frequency analysis of protein targets. **d** KEGG pathway enrichment analysis. The gradual change in color represents the change in probability. **e** GO function analysis. The gradual change in color represents the change in probability

To identify the pharmacological mechanism of JSD on DN, we constructed a network among the active ingredients in JSD, their corresponding targets, and DN-related genes (Fig. 5a). The protein targets retrieved at medium probabilistic confidence scores and the predicted functional partners were presented as nodes in the protein–protein interaction (PPI) network (Fig. 5b). Network nodes indicate protein targets or the relevant genes. By node degree statistics, we found that targets such as Akt, MAPK, IL-6, and VEGF were located in the central network (Fig. 5c), and some markers were also considered targets involved in our in vivo study.

To better understand the functions of JSD, we carried out KEGG pathway enrichment and GO function analysis based on these putative targets. We found that these putative targets not only modulated cell proliferation, apoptosis, growth, and inflammatory response but also fine-tuned phosphatase binding, protein serine/threonine kinase activity, MAPKs, and advanced glycation end products and receptor of glycation end products (AGE-RAGE) signaling pathways (Fig. 5d, e). These results were useful in further and better understanding the mechanisms of JSD in DM or DN.

**Fig. 6:**
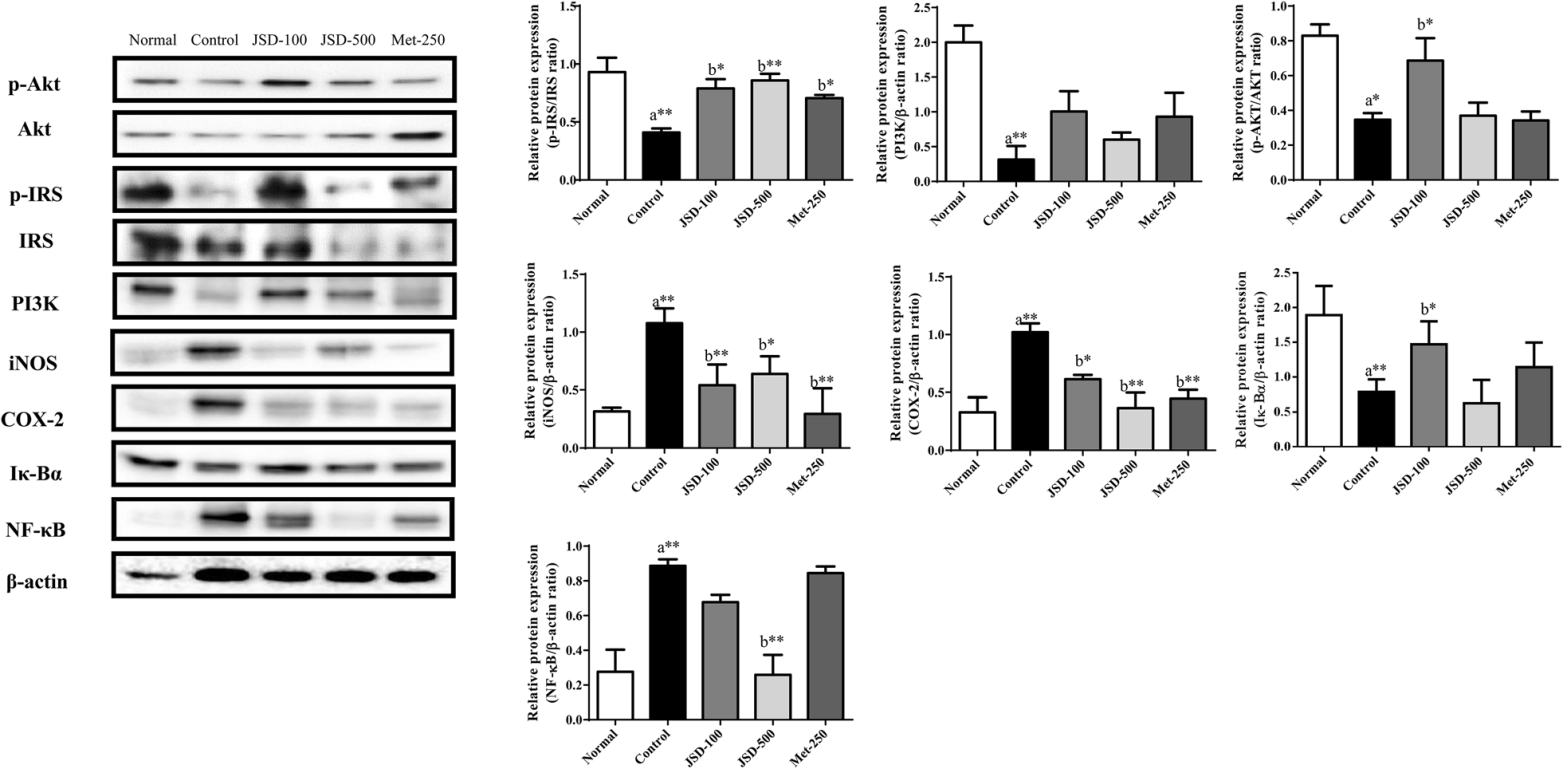
Effects of JSD on the PKCα/PI3K/Akt and NF-κB/α-SMA signaling pathways in the kidney tissues of DN mice. Relative levels of phosphorylated the Akt-Ser473, IRS-Ser307, PI3K p85, iNOS, COX-2, I-κBα, and NF-κB p65 proteins were determined with total and phosphorylated forms or β-actin for normalization determined by western blotting. Normal, normal group; Control, STZ-induced DN control group; JSD-100, 100 mg/kg JSD-administered group; JSD-500, 500 mg/kg JSD-administered group; and Met-250, 250 mg/kg metformin-administered group. Data are expressed as the mean ± S.D. (n = 7). *p < 0.05, and **p < 0.01, the control group vs. the normal group (**a**) and the JSD-100, JSD-500 and Met-250 groups vs. the control group (**b**)

To verify the mechanism of JSD responsible for the inhibition of renal damage in DN, we investigated signaling pathways associated with metabolic disorders, insulin resistance and inflammation by western blot analysis (Fig. 6).

The results showed that the phosphorylation of Akt (*p* < 0.05) and IRS-1 (*p* < 0.01) and the expression of PI3K (*p* < 0.01) were significantly decreased in DN mice compared with normal mice. Compared with the control condition, the administration of JSD significantly increased the phosphorylation of Akt (*p* < 0.05 for low doses) and IRS-1 (*p* < 0.05 for the low dose and *p* < 0.01 for the high dose) and the expression of PI3K. Metformin administration increased the phosphorylation of Akt and IRS-1 and the expression of PI3K in DN mice.

The results showed that the expression levels of iNOS (*p* < 0.01) and COX-2 (*p* < 0.01) were significantly increased in the DN control group compared with the normal group. The administration of JSD significantly decreased the expression of iNOS (*p* < 0.01 for the low dose and *p* < 0.05 for the high dose) and COX-2 (*p* < 0.05 for the low dose and *p* < 0.01 for the high dose) in DN mice. Metformin also significantly inhibited the expression of iNOS (*p* < 0.01) and COX-2 (*p* < 0.01) in kidney tissues.

A significant increase in the expression of I-κBα (*p* < 0.01) and a decrease in the expression of NF-κB (*p* < 0.01) were observed in the control group compared with the normal group. However, the administration of JSD at low (*p* < 0.05 for I-κBα) and high (*p* < 0.01 for NF-κB) doses showed a significant increase in I-κBα expression and a significant decrease in NF-κB expression in the kidney tissues of DN mice.

### Gut microbiota analysis

In the present study, the high dose of JSD improved the symptoms of STZ-induced DN in mice. Therefore, a high dose (500 mg/kg) of JSD was selected as the optimal effective dose for gut microbiota analysis.

First, all data should be quality evaluated via statistical methods. According to the sequence number, sequence length, GC content, Q20 and Q30 quality values, effectiveness and other parameters (Table 4) in each stage, all the data meet the quality assessment requirements. Then, the length of the effective tags in each sample was calculated, as shown in Fig. S2 in Additional file 1).

**Table 4:** Sequencing data for each sample

Sample ID	PE reads	Raw tags	Clean tags	Effective tags	AvgLen (bp)	GC (%)	Q20 (%)	Q30 (%)	Effective (%)
Normal1	79,826	71,406	64,172	62,037	417	53.94	97.8	95.68	77.72
Normal2	79,990	74,027	68,381	63,980	413	54.21	97.87	95.82	79.98
Normal3	79,776	72,191	64,790	62,138	416	53.83	97.92	95.91	77.89
Normal4	80,063	71,608	64,070	62,234	417	53.46	97.75	95.6	77.73
Cont1	79,857	71,232	63,420	60,788	419	53.66	97.82	95.75	76.12
Cont2	80,039	72,767	66,452	63,901	414	54.66	97.73	95.57	79.84
Cont3	79,910	76,987	73,661	70,123	416	52.25	98.0	96.09	87.75
Cont4	79,968	71,690	64,205	61,409	416	53.08	97.73	95.59	76.79
Cont5	80,087	73,036	66,707	65,195	415	54.33	97.92	95.85	81.41
Cont6	79,946	69,589	60,860	59,843	421	53.91	97.82	95.7	74.85
JSD1	79,697	73,088	66,970	64,768	416	54.22	97.81	95.7	81.27
JSD2	80,075	73,609	67,315	63,416	415	53.59	97.88	95.85	79.2
JSD3	79,844	72,784	66,551	64,925	417	53.9	97.8	95.66	81.31
JSD4	79,995	74,039	68,474	66,006	415	54.11	97.77	95.63	82.51
JSD5	80,121	74,950	69,798	67,816	416	53.18	97.85	95.78	84.64
JSD6	80,349	74,416	69,006	66,212	415	53.65	97.72	95.57	82.41
Met1	79,977	72,743	66,571	63,895	416	53.38	97.75	95.57	79.89
Met2	79,879	67,384	57,445	55,569	413	53.77	97.59	95.36	69.57
Met3	79,823	71,404	63,046	61,126	417	54.16	97.86	95.77	76.58

An operational taxonomic unit (OTU) is an artificial identification of a taxon (strain, species, genus, group, etc.) for analysis in phylogenetic research or population genetics studies. Generally, if the similarity between sequences is higher than 97%, the sequence can be defined as an OTU, and each OTU corresponds to a representative sequence. In our study, based on the taxonomy databases of Silva (bacteria) and UNITE (fungi), taxonomic annotation of OTUs was carried out, and the number of OTUs in each sample as determined by clustering was shown in Fig. S3 in Additional file 1).

A Venn diagram (Fig. S4 in Additional file 1) can be used to show the number of common and unique OTUs between different samples in different groups. Combined with the species represented by OTUs, common microorganisms in different environments can also be identified. In our study, there were 399 shared OTUs in the 4 groups and 461, 471, 469 and 423 unique OTUs in the normal, control, JSD and metformin groups, respectively.

**Fig. 7:**
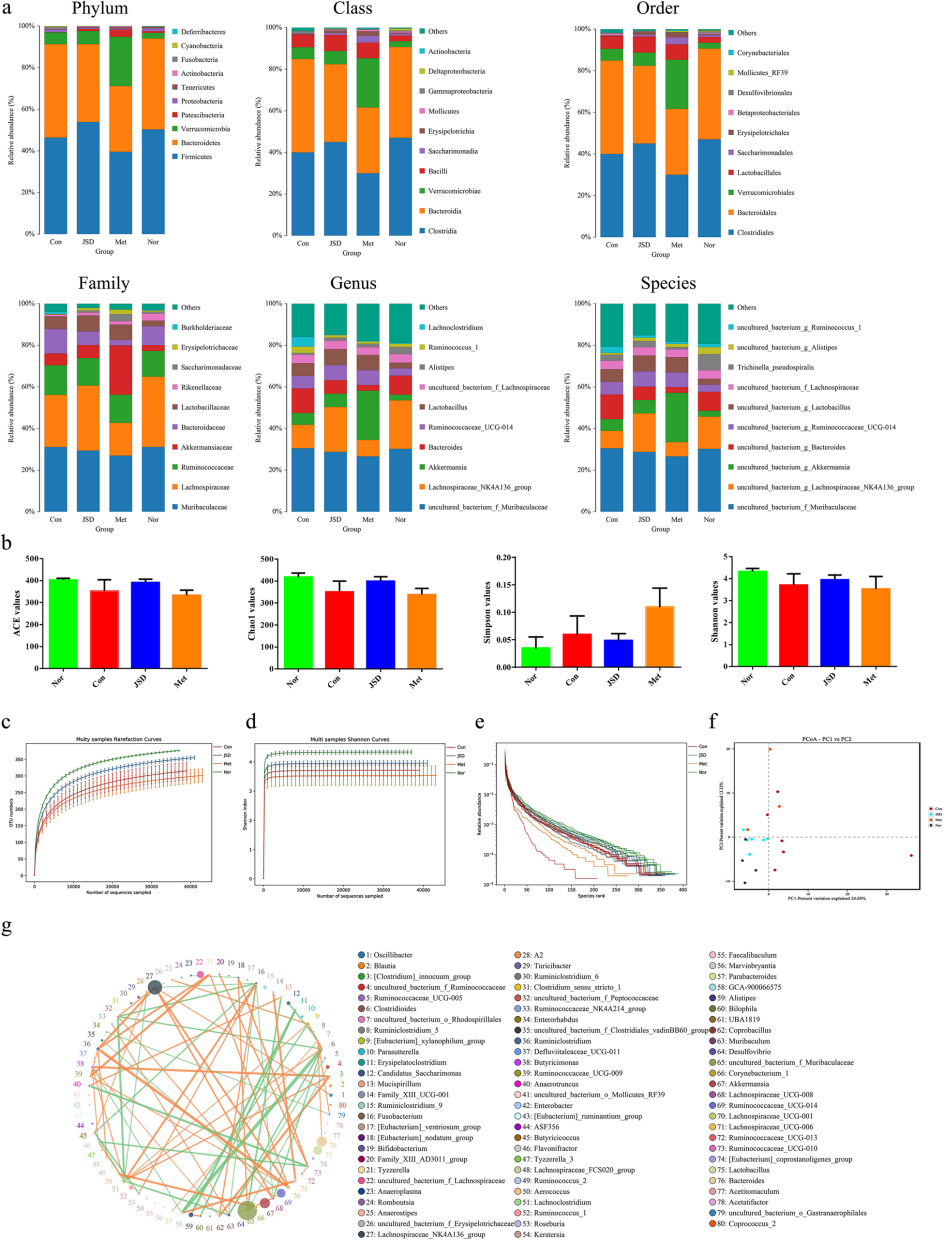
Gut microbiota analysis. **a** Information on the phylum, class, order, family, genus, and species of each group. **b** Measurement indexes for alpha diversity analysis. **c** Rarefaction curve. **d** Shannon index curve. **e** Rank abundance curve. **f** Principal coordinates analysis. **g** Correlation analysis for gut microbiota; Nor, normal group; Con, STZ-induced DN control group; JSD, 500 mg/kg JSD-administered group; and Met, 250 mg/kg metformin-administered group

As shown in Table 5, after deleting the OTU with very low abundance (species abundance less than 0.005%), the final OTU list was obtained, the tags of species in each sample were counted, and the number of species types at each level in each sample was shown in Table 6. The species distribution information was further annotated by the relative abundance value, and the relative abundance for the top ten of each group at the phylum, class, order, family, genus, and species levels was shown in Fig. 7a.

**Table 5:** The number of OTUs in each sample at different levels

Sample	Kingdom	Phylum	Class	Order	Family	Genus	Species
Normal1	43,430	43,430	43,430	43,430	43,430	43,430	43,430
Normal2	37,040	37,040	37,040	37,040	37,040	37,040	37,040
Normal3	41,878	41,878	41,878	41,878	41,878	41,878	41,878
Normal4	42,832	42,832	42,832	42,832	42,832	42,832	42,832
Cont1	45,053	45,053	45,053	45,053	45,053	45,053	45,053
Cont2	41,532	41,532	41,532	41,532	41,532	41,532	41,532
Cont3	62,026	62,026	62,026	62,026	62,026	62,026	62,026
Cont4	39,210	39,210	39,210	39,210	39,210	39,210	39,210
Cont5	45,779	45,779	45,779	45,779	45,779	45,779	45,779
Cont6	47,511	47,511	47,511	47,511	47,511	47,511	47,511
JSD1	45,553	45,553	45,553	45,553	45,553	45,553	45,553
JSD2	41,009	41,009	41,009	41,009	41,009	41,009	41,009
JSD3	48,956	48,956	48,956	48,956	48,956	48,956	48,956
JSD4	47,620	47,620	47,620	47,620	47,620	47,620	47,620
JSD5	50,598	50,598	50,598	50,598	50,598	50,598	50,598
JSD6	46,544	46,544	46,544	46,544	46,544	46,544	46,544
Met1	50,688	50,688	50,688	50,688	50,688	50,688	50,688
Met2	43,264	43,264	43,264	43,264	43,264	43,264	43,264
Met3	47,571	47,571	47,571	47,571	47,571	47,571	47,571

**Table 6:** Number of taxon in each sample at different levels

Sample	Kingdom	Phylum	Class	Order	Family	Genus	Species
Normal1	1	9	15	20	33	85	87
Normal2	1	9	14	16	28	78	80
Normal3	1	10	16	22	36	84	86
Normal4	1	9	15	17	31	82	84
Cont1	1	10	16	22	35	83	86
Cont2	1	10	16	21	33	84	85
Cont3	1	9	15	21	34	72	73
Cont4	1	10	16	21	35	86	88
Cont5	1	10	15	19	32	78	80
Cont6	1	9	14	21	36	83	85
JSD1	1	9	15	21	34	84	86
JSD2	1	9	15	22	33	85	88
JSD3	1	9	14	19	33	83	85
JSD4	1	10	15	22	36	86	88
JSD5	1	9	14	20	32	82	84
JSD6	1	10	16	20	36	90	92
Met1	1	10	16	20	33	78	78
Met2	1	9	14	20	35	88	91
Met3	1	9	14	19	31	78	79

The species abundance and diversity of a single sample is represented by alpha diversity, and the Chao1, Ace, Shannon and Simpson indexes are four common measurement indexes. Chao1 and Ace values can reflect the species abundance and number, and species diversity can be indicated by the Shannon and Simpson values. A higher species diversity can be shown with a high Shannon value and a low Simpson value. In our study, there were low ACE, Chao1 and Shannon values and high Simpson values in the STZ-induced control group compared with the normal group (Fig. 7b). After oral JSD administration, the ACE, Chao1 and Shannon values increased, and the Simpson value decreased. However, there was no obvious change in those indexes in the metformin treatment group. Moreover, those index results can be further verified by the results of the rarefaction curve (Fig. 7c), Shannon index (Fig. 7d) and rank abundance curve (Fig. 7e). Therefore, JSD has a significant callback effect on bacterial diversity.

To compare the similarity of species diversity between different samples in different groups, principal coordinates analysis (PCoA) (Fig. 7f) was performed by beta diversity analysis. In the PCoA analysis, the points of the JSD group were widely distributed between the normal and DN control groups, which means that JSD can actively recover the structure of gut microbiota to that observed in the control group. However, the metformin treatment group did not show a consistent positive regulatory effect compared with the JSD group.

The Metastats method was used to search for biomarkers with significant differences (LDA score > 4). The following significant differences in bacteria were observed between the normal and DN control groups: Patescibacteria and Deferribacteres at the phylum level; Saccharimonadia, Actinobacteria and Deferribacteres at the class level; Saccharimonadales, Corynebacteriales, Deferribacterales and Enterobacteriales at the order level; Saccharimonadaceae, Atopobiaceae, Rikenellaceae, Corynebacteriaceae, Deferribacteraceae, Enterobacteriaceae and Aerococcaceae at the family level; and Desulfovibrio, Lactobacillus, Muribaculum and Ochrobactrum at the genus level. After JSD oral administration, the following significant changes in the gut microbiota between the control and the JSD treatment groups were observed: Alphaproteobacteria at the class level; Atopobiaceaem at the family level; and Acetatifactor, Butyricicoccus, Kerstersia, Peptococcus and Coriobacteriaceae_UCG-002 at the genus level. These results revealed that JSD treatment significantly affected the gut microbiota.

Correlation analysis was performed and visualized with a network diagram (Fig. 7g). f_Muribaculaceae, Lachnospiraceae_NK4A136_group, Akkermansia, Bacteroides, Lactobacillus, Ruminococcaceae_UCG-014 and f_Lachnospiraceae had high frequencies in the interconnected floras.

## Discussion

STZ is a naturally occurring alkylating antineoplastic agent that is particularly toxic to insulin-producing beta cells in the pancreas and thus induces experimental diabetes mellitus (DM) and diabetic complications, including DN [9]. In our study, we established DN in mice by STZ injection once per day for 3 consecutive days while the mice were maintained on a normal diet. DN mice developed systematic and serious histopathological damage in the kidney, as well as typical diabetic symptoms, such as hyperglycemia, polydipsia, polyuria, and severe weight loss.

JSD is a representative prescription consisting of three herbs and is clinically used to treat patients with DM and diabetic complications, including DN, in traditional Chinese and Korean medicine [15]. Although the individual drugs in JSD have various effects, such as anti-inflammatory, antioxidant [16, 17] and antidiabetic effects [18–20], as demonstrated by in vitro and in vivo experiments, there limited scientific evidence shows the efficacy of JSD on DM and DN. However, in the HPLC analysis, liquiritin and ammonium glycyrrhizate in Liquorice and aloe-emodin, rheinic acid, rheum emodin, chrysophanol, and emodin-3-methyl ether in Rhubarb in JSD were identified as the main compounds in JSD. This result can help to control the quality of JSD and to facilitate further studies, such as pharmacodynamics research.

It has been reported that typical symptoms of DN are hyperglycemia, polydipsia, urorrhagia, and glucose, fat, and protein metabolism imbalance [21]. Cr and UN levels in serum and urine are considered common markers of renal function [22]. The administration of JSD at both low and high doses significantly decreased the increase in food and water intake, urine volume, glucose and triglyceride levels, and urinary albumin excretion in DN mice. In addition, JSD significantly increased insulin release and normalized creatinine clearance through the inhibition of the morphological destruction of kidney tissues. These results indicate that JSD can improve DN symptoms through the inhibition of abnormal changes in physiological and serological characteristics and structural damage to liver and kidney tissues. Regarding the constituents of JSD, Rhubarb is a herb commonly used in traditional medicine for diarrhea, removing stagnancy, heat clearing, fire draining, detoxicating, promoting blood circulation, and removing blood stasis; therefore, this herb is useful for clearing heat and relieving turbidity in DN. The literature [14, 23, 24] has reported that this herb has good antidiabetic effects. Liquorice is a sweet-natured herb and is clinically applied as a classical adjuvant herb in many prescriptions. Modern pharmacological studies have shown that this herb has synergistic effects with other herbs on DM [14, 25]. The effects of each herb in JSD can provide theoretical references regarding the effect of JSD on DM and DN.

The metabolic dysregulation in DM may eventually cause tissue damage. The liver is the main organ of endogenous glucose generation, fatty acid removal, and insulin metabolism, which plays a key role in the adjustment of lipid metabolism in DM [26]. Kidney function is closely related to the progression of DM and diabetic complications, and severe DN is the second most common metabolic disease associated DM [27]. According to traditional medicine, the liver and kidney are homologous. Although their structure and functions are different, their origins are the same, which means that their physiological and pathological characteristics are closely related to each other [28]. In this study, we extensively investigated the mechanisms responsible for the therapeutic effects of JSD on DN, particularly focusing on liver and kidney dysfunction. Hyperglycemia induces renal injury through multiple pathways, including insulin resistance, glucose metabolism, hemodynamic disorders, inflammation, and fibrosis [29]. After long-term and chronic kidney damage in DN, incomplete tubular recovery leads to renal fibrosis and destroyed renal function. In this study, we found that JSD effectively inhibited renal fibrosis in STZ-induced DN mice through the inhibition of the PKC-α/TGF-β1/α-SMA signaling pathways. In morphological observations, we also found that JSD administration decreased the increase in glomerular volume, nodular sclerosis, mesangial stromal hyperplasia, glomerular basement membrane thickness, hypertrophy (H&E stain), mesangial matrix expansion (PAS stain), and fibrosis intensity in response to the accumulation of extracellular matrix proteins (M-T stain) in DN mice. These results indicate that JSD can protect kidney tissues against DN-induced damage.

To further identify the pharmacodynamic mechanism of JSD in DN, we constructed a network of the active ingredients in JSD, their corresponding targets, and DN-related genes by network pharmacological analysis. Interestingly, we found consistency in the results between our in vivo study and the network pharmacological prediction. In our analysis, phosphatase binding and protein serine/threonine kinase activity involving Akt phosphorylation and IRS-1 and PI3K expression were core targets in signal transduction, metabolism regulation [30], cell proliferation [31], inflammation [32], and insulin resistance [33] in DN. MAPKs are important transmitters of signaling cascades from the cell surface to the nucleus, and I-κBα/NF-κB are the main messengers in the MAPK pathway [34]. The activation of MAPKs regulates a variety of important cellular physiological and pathological processes, including cell growth, differentiation, stress adaptation to the environment, and inflammatory response in cells [35]. Moreover, the pharmaco-mechanism of JSD responsible for renal protection predicted by network pharmacological analysis was further verified by western blot analysis, and the changes in inflammation- and insulin resistance-related signaling pathways in the kidney tissues of DN mice. It has been previously reported that the phosphorylation of IRS-1 induces a reduction in the activities of enzymes in the PI3K/Akt pathway, a downstream signaling component of glucose metabolism [23]. PI3K/Akt phosphorylation is also associated with the enhancement of high glucose-stressed apoptosis in STZ-induced DN [24]. In addition, Akt appears to positively regulate the excessive production of inflammatory mediators, such as iNOS and COX-2, through the activation of the I-κB/NF-κB pathway in DN [36]. Therefore, the activation of the PI3K/Akt/IRS-1 and I-κB/NF-κB pathways induces a decrease in the activities of enzymes, such as phosphatase and protein serine/threonine kinase, which are crucial in the pathogenesis of renal failure [37]. In this study, we found that JSD administration significantly reduced the expression of iNOS and COX-2 in the kidney tissues of DN mice through inhibition of the I-κB/NF-κB pathway, as well as phosphorylation of the PI3K/Akt pathway. This finding indicates that the preventive effects of JSD on the renal damage induced by fibrosis and inflammation are due to the downregulation of the PI3K/Akt and I-κB/NF-kB signaling pathways in DN. However, from the network pharmacological analysis, we collected a large amount of information about the pharmaco-mechanisms of JSD in DN and found that our signaling targets related renal damage in DN mice were well matched to the functional targets in the network pharmacology analysis, suggesting that we can predict the functions of JSD and the molecular mechanism in DN. These predictions will be explored in a further study about other targets, functions, or signaling pathways in the network pharmacology analysis.

The intestinal flora is closely related to human health, and the imbalance of intestinal flora may be involved in the occurrence and development of DM, while metabolic environment changes in people with diabetes may affect the composition and function of the intestinal flora [38]. After testing anti-DN potential, the mechanism of JSD was assessed by gut microbiota analysis. Rhubarb, as the primary drug in JSD, has been recorded in many studies to have anti-inflammatory effects and protect against chronic diseases by regulating intestinal flora structures [39, 40]. The results of multivariate analysis also showed that JSD can adjust the flora structures, especially at the class, family and genus levels, which further involves the inflammatory response and metabolic disorders in DM or DN. Alphaproteobacteria is a marker bacterium that distinguishes normal and diabetic patients [41]. Our results showed a significant change in the class Alphaproteobacteria between the control and JSD-treated groups. Acetatifactor is an important bacterium involved in fatty acid metabolism. The regulation of fatty acids in metabolism is bidirectional; fatty acid regulation can increase fat deposition by increasing energy utilization and can improve metabolic disorders by reducing the inflammatory response and regulating the hormone secretion of intestinal endocrine cells [42]. The imbalance of Peptococcus can involve the inflammatory response and cause the infection of tissues and organs [43]. Moreover, Coriobacteriaceae can promote the conversion and absorption of the medicinal compounds in JSD [44].

Although some adverse reactions may be caused by long-term use, metformin is the most classic antidiabetic drug used for the treatment of T2DM [45]. In our study, the administration of JSD and metformin in STZ-induced DN mice showed a similar therapeutic effect, which provides valid evidence that JSD, a traditional prescription, could be a good candidate and alternative medicine for DN treatment with effects similar to the Western medicine metformin.

Traditional and alternative medicine has a long history of protecting the human healthcare system by the use of herbal prescriptions in Asia. Herbal prescriptions combine a large number of active chemicals. Owing to the complexity of the chemical composition, the mechanisms of action of medicinal herbs or decoctions are still unclear. To our knowledge, our present study is the first report of the anti-DN effects of JSD in a mouse model. We identified the stable existence of chemical substances both in JSD and single herbs to ensure the quality of JSD, but some changes in the chemical compositions of individuals drugs may have occurred during the decocting process. Our study on the classical pathways of JSD in DN identified by network pharmacological analysis can offer further information for understanding the function of potent chemicals and mechanisms in vivo, such as pharmacokinetic or metabonomic analysis. We also did not offer additional explanations on the intrinsic relevance between changes in pathology and the diversity of intestinal flora. These limitations and deficiencies need to be explored in further studies.

## Conclusions

The administration of JSD at 100 and 500 mg/kg to STZ-induced DN mice for 4 weeks improved physiological and serological imbalances and renal and liver damage. The network pharmacology paradigm revealed that JSD acted through the regulation of the PKCα/PI3K/Akt and NF-κB/α-SMA signaling pathways in DN mice. Gut microbiota analysis can help to discover the pharmaco-mechanisms of JSD that have a significant effect on bacterial diversity and community structures in DN.

## Supplementary information

**Additional file 1: Figure S1.** This section includes the supplementary figures of Venn intersection targets diagram. **Figure S2.** Effective Tags length distribution graph for each sample. **Figure S3.** Distribution of the number of OTUs in each sample. **Figure S4.** Shared and unique OTUs in the normal, control, JSD and metformin groups.

## Data Availability

The datasets used and/or analyzed during the current study are available from the corresponding author on reasonable request.

## Abbreviations

Akt: Protein kinase B
BATMAN-TCM: A Bioinformatics Analysis Tool for Molecular mechanism of Traditional Chinese Medicine
BW: Body weight
JSD: Jowiseungki decoction
CAM: Complementary and alternative medicines
COX-2: Cyclooxygenase-2
Cr: Creatinine
DAG: Diacylglycerol
DL: Drug-likeness
DM: Diabetes mellitus
DN: Diabetic nephropathy
ECM: Extracellular matrix
FBG: Fasting blood glucose
GBM: Glomerular basement membrane
H&E: Hematoxylin–eosin staining
HDL-C: High density lipoprotein cholesterol
iNOS: Inducible nitric oxide synthase
IκBα: Nuclear factor of kappa light polypeptide gene enhancer in B‑cells inhibitor, alpha
MA/UCREA: Microalbumin/urine creatinine
MA: Microalbumin
NF‑κB: Nuclear factor kappa‑B
OB: Oral bioavailability
OMIM: The Online Mendelian Inheritance in Man
OTUs: Operational taxonomic units
p‑Akt: Phosphorylated protein kinase B
PAS: periodic acid-Schiff staining
PCA: Principal component analysis
PI3K: Phosphatidylinositol 3‑kinase
PKC: Protein kinase C
p‑PI3K: Phosphorylated phosphatidylinositol 3‑kinase
TCM: Traditional Chinese medicine
TCMSP: Traditional Chinese medicine systems pharmacology
TG: Triglyceride
TGF-β1: Transforming growth factor β-1
TKM: Traditional Korean medicine
UN: Urea nitrogen
α-SMA: alpha-Smooth muscle actin
