# Supplementary material for: Jowiseungki decoction affects diabetic nephropathy in mice through renal injury inhibition as evidenced by network pharmacology and gut microbiota analyses
Source: Chin Med. 2020 Mar 12;15:24. doi: 10.1186/s13020-020-00306-0 (PMC7066842; doi:10.1186/s13020-020-00306-0)
Supplement: Supplementary file 1 — Additional file 1: Figure S1. This section includes the supplementary figures of Venn intersection targets diagram. Figure S2. Effective Tags length distribution graph for each sample. Figure S3. Distribution of the number of OTUs in each sample. Figure S4. Shared and unique OTUs in the normal, control, JSD and metformin groups. [file 13020_2020_306_MOESM1_ESM.doc]

Additional file 1


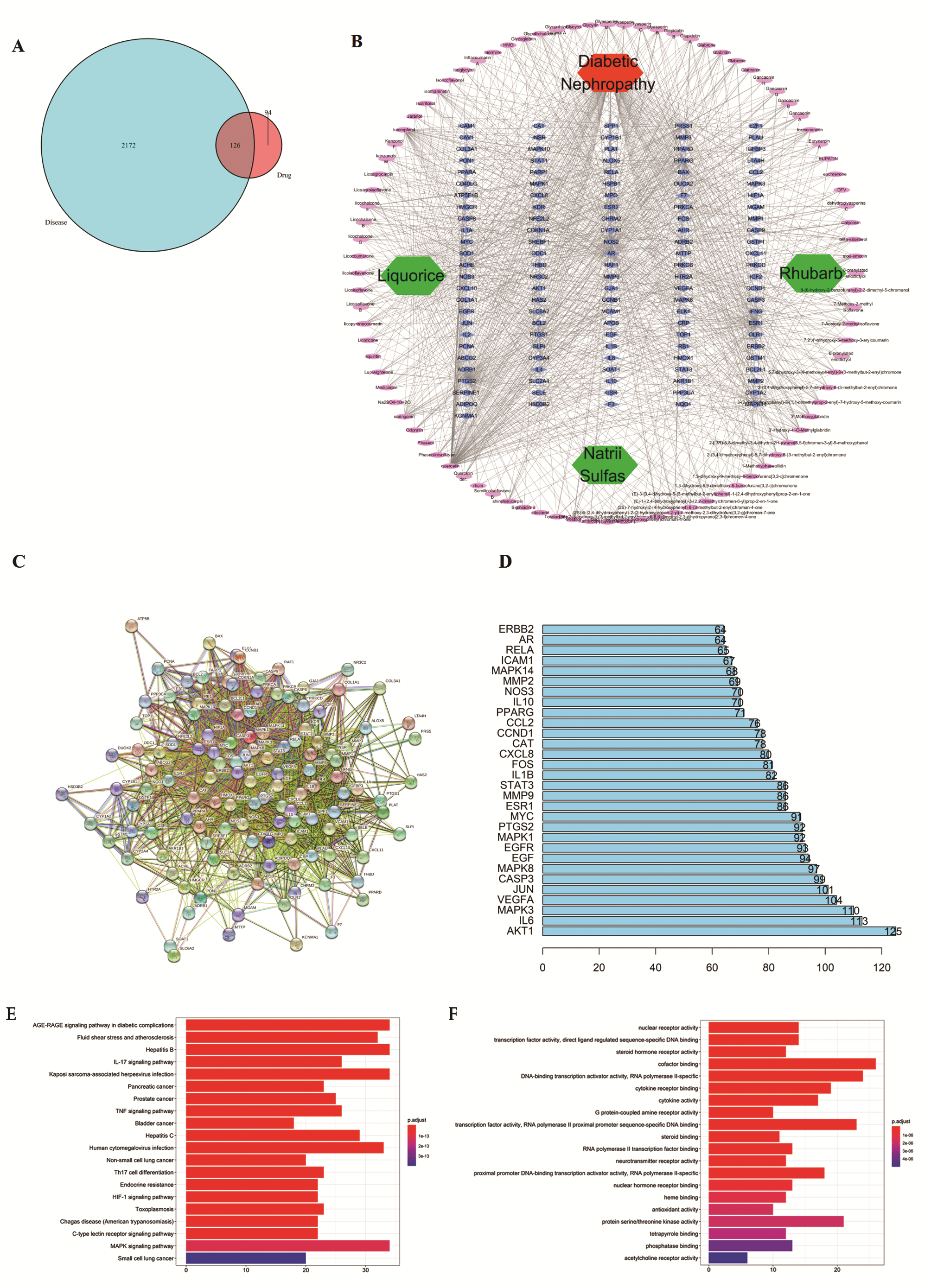


Additional Figure S1. Venn intersection targets diagram





Additional Figure S2. Effective Tags length distribution graph for each sample


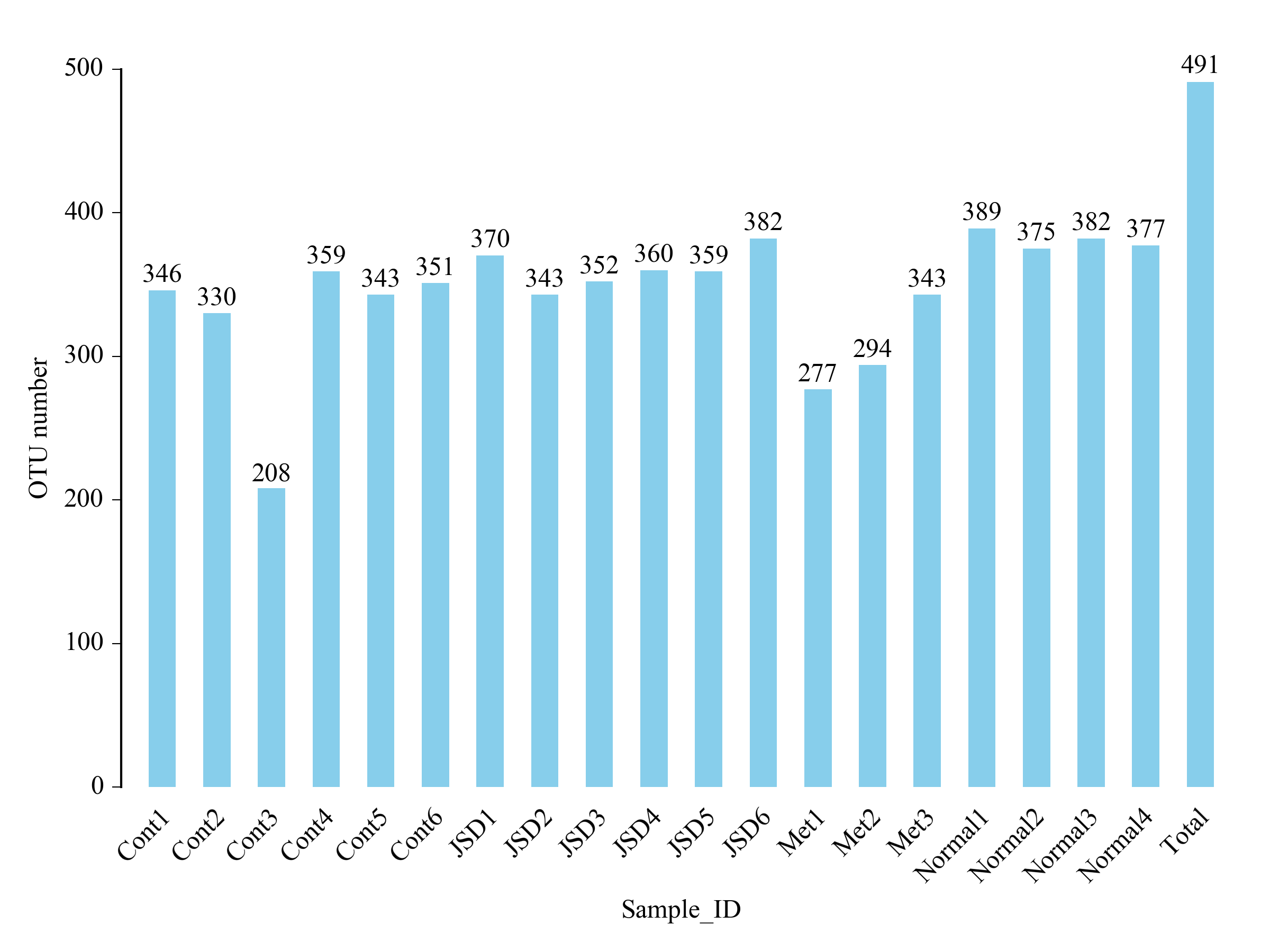


Additional Figure S3. Distribution of the number of OTUs in each sample


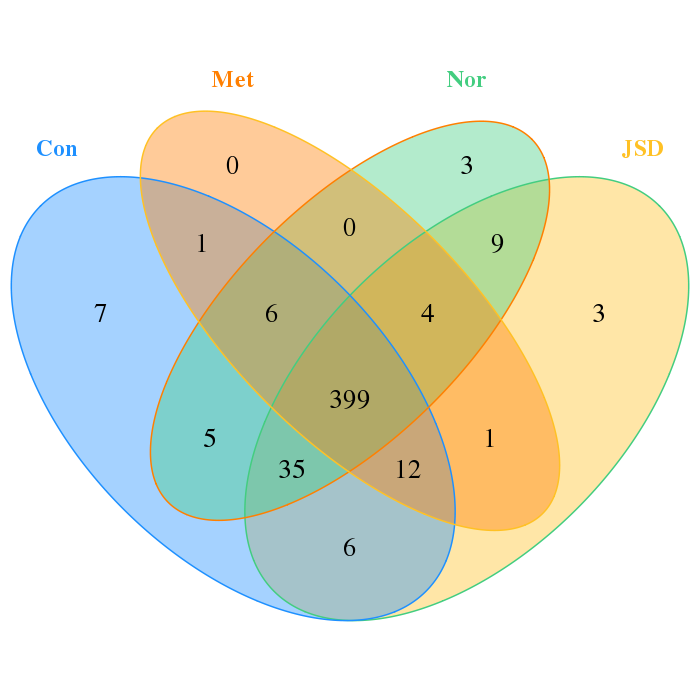


Additional Figure S4. Shared and unique OTUs in the normal, control, JSD and metformin groups.
